# Using 2D integral breadth to study plastic relaxation in a quasi-lattice-matched HgCdTe/CdZnTe heterostructure

**DOI:** 10.1107/S1600576722008184

**Published:** 2022-10-01

**Authors:** Xavier Biquard, Aymeric Tuaz, Philippe Ballet

**Affiliations:** a Université Grenoble Alpes, CEA, IRIG, MEM, NRS, 38000 Grenoble, France; b Université Grenoble Alpes, CEA, LETI, 38000 Grenoble, France; Oak Ridge National Laboratory, USA, and North Carolina State University, USA

**Keywords:** HgCdTe, elastic behaviour, plastic deformation, thin films, dislocations

## Abstract

Micro-Laue diffraction has been used to record cross-section profiles on a quasi-lattice-matched HgCdTe/CdZnTe heterostructure as a function of the stress induced by a flexion machine. The heterostructure may be decomposed into four different regions according to depth. Sufficiently far from the interface, the CdZnTe substrate is undisturbed by the HgCdTe layer, while the region situated 10 µm beneath the interface presents an in-plane lattice parameter adjustment to the +0.02% mismatched layer. The layer has a 2 µm critical thickness and, beyond, misfit dislocations induce a large peak broadening whose main direction changes with depth. The same occurs over the whole heterostructure once flexion-induced plastification has started. Consequently, the usual full width at half-maximum or integral breadth is no longer relevant, and only a newly defined and rotationally invariant 2D integral breadth correctly measures the plastification-induced peak broadening. Taking into account only the critical thickness region, a 15.1 ± 0.7 MPa tensile HgCdTe elastic limit was measured, slightly overestimated because of the initial compressive layer stress. It was observed that the plastic onset of the substrate perfectly matches the elastic limit of the layer, despite the fact that the substrate elastic limit is expected to be four times higher: a striking demonstration of the propagation of threading dislocations. The ‘plastification easiness’ is found to be 2.4 times smaller deep inside the substrate than in the layer critical thickness region, while in the substrate lattice adjustment region, the plastification easiness goes from the substrate to the layer value with a 22–25 MPa transition interval. This novel method using the 2D integral breadth allows for easy critical thickness measurement as well as precise plastic onset determination and plastification easiness assessment. It is a quite general method, since it may be applied to the vast class of epitaxial layers for which the critical thickness is larger than the micro-Laue beam size (currently 250 nm).

## Abstract 2

The newly defined and rotationally invariant 2D integral breadth correctly measures plastification-induced peak broadening during micro-Laue diffraction experiments, and allows for both critical thickness and plastic onset measurements. Applied to the quasi-lattice-matched HgCdTe/CdZnTe heterostructure and taking into account the critical thickness only, it showed that the plastic onset of the rigid substrate perfectly matches the elastic limit of the smooth layer: a striking demonstration of the propagation of threading dislocations.

## 1. Introduction

HgCdTe is recognized as one of the best materials for IR detection application. Its unique ability to detect IR photons at various wavelengths by simple adjustment of the Cd/Hg alloy fraction enables the design of IR devices dedicated to different bands or even multiple bands simultaneously (Tennant *et al.*, 2001 ▸; Ballet *et al.*, 2004 ▸; Smith *et al.*, 2004 ▸). Choosing a specific alloy composition can be done with very little change in lattice constant, and all HgCdTe material can in principle be grown on a perfectly lattice matched CdZnTe substrate given careful determination of the Zn content.

However, from a practical point of view, exact lattice matching has proved difficult to achieve because of the ternary nature of the two materials, HgCdTe and CdZnTe. Zinc dispersion within the CdZnTe substrate will inevitably result in lattice mismatch in the low 10^−4^ range. But, because efficient IR absorption requires a large thickness of HgCdTe, dislocation generation is likely to appear at some point during growth or later during the microfabrication processing of the operating devices. This idea can be further documented, considering the low elastic limit of HgCdTe. This elastic limit has been measured to be in the 10–20 MPa range, depending slightly on the tensile or compressive nature of the stress (Ballet *et al.*, 2013 ▸). A rapid calculation of the stress induced by the differences in the coefficient of thermal expansion of CdTe and Si, used as the read-out layer of IR detectors, gives a value of 20 MPa when the device is cooled to 100 K, which is close to the operating temperature for most applications.

From this perspective, it appears quite important to investigate further the plastic onset in this material system together with the mechanisms underlying the plastic relaxation. In order to experimentally access the onset of relaxation, we decided to carry out a flexion experiment while performing a full micro-Laue investigation of the stress along the growth direction in both layer and substrate. Micro-Laue in cross-section geometry has proved to be very efficient in extracting strain profiles in HgCdTe heterostructures with unprecedented accuracy (Biquard *et al.*, 2021 ▸) and also in full mapping of the deformation fields caused by numerous microfabrication steps (Tuaz *et al.*, 2017 ▸).

In this article, we present the first local measurement of stress during a flexion experiment in the HgCdTe/CdZnTe system, thus fully revealing the plastic relaxation mechanisms. We also define a quite general 2D integral breadth notion that enables precise measurement of the critical thickness, the plastification onset and the ease of plastification. The experiment has been conducted on the French CRG BM32 micro-Laue beamline of the European Synchrotron Radiation Facility (ESRF) in Grenoble, France.

## 2. Sample description

Our two samples (test and data) are as-grown samples made of a single 4.6 µm-thick epitaxial Hg_0.3_Cd_0.7_Te layer of constant alloy composition grown on a ∼700 µm-thick (211)B CdZnTe substrate, with a Zn fraction close to 4%. The layer thickness and exact alloy composition were determined post-growth by Fourier transform IR transmission. The CdZnTe substrates were wet-etched to remove residual polishing damage and *in situ* thermally de-oxidized prior to epitaxy. The growth was achieved by molecular beam epitaxy, ensuring an abrupt interface between layer and substrate, as confirmed by the composition profiles obtained from secondary ion mass spectrometry depth profiling. No buffering is done here, so that the HgCdTe layer is directly grown on the surface of the substrate. During the growth, the Hg, Te and Cd fluxes were kept constant, the latter being carefully adjusted to provide the desired alloy composition. The growth was performed at low temperature (453 K) and, because of the very low sticking coefficient of Hg, a large Hg/Te flux ratio of the order of several 100:1 was maintained during the growth. We measured with high-resolution X-ray diffraction (HRXRD) a +0.02% mismatch between layer and substrate, indicating that the layer is coherently compressively strained onto the substrate (Ballet *et al.*, 2013 ▸). For our micro-Laue cross-section analysis, we cleaved the samples along the (011) plane to make sure that the surface was cleaved perpendicularly to the interface.

Both HgCdTe and CdZnTe are of the zincblende crystal structure with Te occupying site *B*, while either Cd, Hg or Zn occupies site *A* in proportion to their abundances. The selection rules imply that the Miller indices *h*, *k* and *l* are all of the same parity with a specific property. When *h* + *k* + *l* = 4*n*, the diffraction intensity is proportional to the sum *f*_*A*_ + *f*_*B*_ of atomic factors, while when *h* + *k* + *l* = 4*n* + 2, the intensity is proportional to the difference |*f*_*A*_ − *f*_*B*_| of the atomic form factors. Consequently, *h* + *k* + *l* = 4*n* + 2 peaks are weak while others are strong.

## 3. Experimental

**Figure 1:**
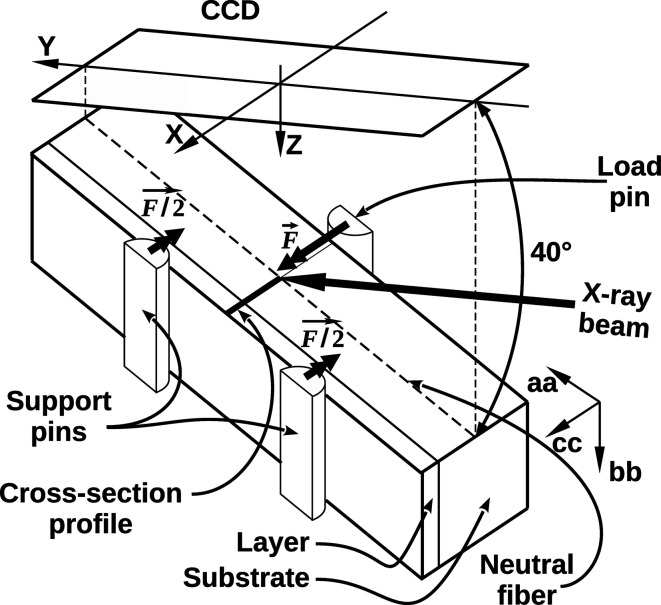
Experimental setup showing on top the CCD camera that intercepts diffraction peaks and its (*X*, *Y*, *Z*) basis. The sample is held by the flexion machine, schematically represented by its load and support pins, the ensemble being rotated 40° around *X*. The X-ray beam impacts along the cross-section profile situated in the tensile zone at the opposite side of the load pin. The sample (**aa**, **bb**, **cc**) basis is also shown, with **cc** being the [211] growth direction, equivalent to *X*. Direction **bb** corresponds to the [011] direction since the cleaved surface is perpendicular to the interface, while the flexural stress is applied along the direction **aa**, corresponding to [11 1].

The micro-Laue setup implemented on the French CRG BM32 beamline at the ESRF enables a white beam with all energies ranging from 5 to 23 keV to be focused down to a sub-micrometre diameter, close to 750 nm at the time of this experiment and nowadays smaller than 250 nm. Diffraction peaks are intercepted upwards with a 16 bit 2048 × 2048 ø165 mm Mar CCD camera, while Hg fluorescence is collected using an energy-resolved detector (Ulrich *et al.*, 2011 ▸). Samples were cleaved as bands of 15 × 4 mm and were positioned inside a commercial Proxima flexion machine from MicroMecha. It was equipped with a three-point bend fixture made of two support pins and a central load pin with a 100 N range strength gauge of ±0.5 N absolute accuracy including both hysteresis and linearity. To avoid damaging the layer, the two support pins are positioned on the layer side while the load pin is on the back-side of the substrate. The flexion machine was fixed onto a holder made of three perpendicular fine mechanical positioning stages of 100 nm resolution equipped with close-loop encoders, the ensemble being rotated at 40° relative to the X-ray direction (see Fig. 1 ▸).

The X-ray beam was aligned with the central load pin so that – for any bending moment – we were able to realize a cross-section profile by recording successive 10 s-duration CCD images at different depths relative to the substrate/layer interface (depth = 0 by convention, positive towards the substrate). The Hg fluorescence profile was fitted with a rectangular function whose width represents the layer thickness, convoluted with a pseudo-Voigt function to represent the beam shape, the step-down position tracking the interface position. We coherently measured a layer thickness of 4.68 ± 0.17 µm and an X-ray beam full width at half-maximum (FWHM) of 700 ± 60 nm. Cross-section profiles were recorded in three parts: the first part focuses on the layer–substrate interaction and goes from above the surface throughout the layer to 5 µm inside the substrate with 0.5 µm steps. The second part focuses on the substrate and goes from depth 6 to 50 µm in 2 µm steps. Finally, the third part is a single, very deep 350 µm measurement (neutral fibre) that will later serve as an unstrained reference.

Making use of an optical microscope equipped with a high numerical aperture apochromatic 50× lens achieving a 300 nm resolving power, we translated our 15 mm-long samples between the 6 mm span support pins until we found a step-free cleaved zone in which to conduct the experiment. The support pins are 5 mm wide so that they completely transfer the bending moment to the 4 mm-wide sample.

The flexural stress experienced inside the sample decreases with depth from the surface, where it is maximal, until the neutral fibre, where it is always zero. The maximum flexural stress σ_f_ experienced by outer fibres at the midpoint for a rectangular cross section is given by the classical formula σ_f_ = (3*L*/2*bh* ^2^)*F*, where *F* (N) is the measured strength on the load pin while *L* (support span), *b* (width of the sample) and *h* (sample thickness) define the proportionality factor. Numerically, we get σ_f_ (MPa) = 4.0*F* (N).

As we must be sensitive to any spontaneous drop in strength because this will signal the elastic-to-plastic transition, the flexion machine cannot work in the usual constant strength mode. It is rather operated in the constant deflection mode, where the sample (central) deflection is adjusted and kept constant thanks to an optical 20 nm resolution encoder, while strength evolution on the load pin is measured.

**Figure 2:**
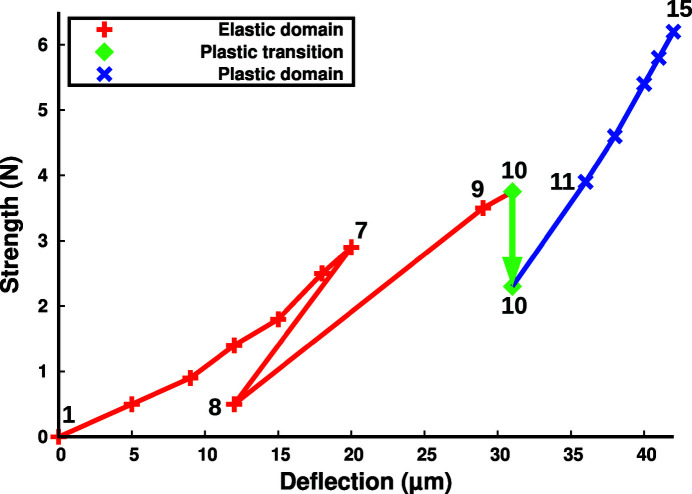
Series of 15 deflection values used and their corresponding strength on the data sample. Deflections 1 to 8 constitute the elastic round trip, while 1 to 9 represent the elastic domain. Spontaneous plastic stress relaxation occurs at deflection 10, causing the strength to drop from 3.75 to 2.3 N. Deflections 11 to 15 represent the plastic domain and at deflection 15 all recorded peaks clearly display a plastic deformation (except at neutral fibre of course).

Making use of the test sample (see the supporting information, Section 7.1), we found that the plastic relaxation occurs in the 27–32 µm deflection range. Therefore, for the data sample, we decided to first record a series of profiles in the elastic domain by limiting the deflection excursion. We went up to a 20 µm deflection (numbers 1 to 7, refer to Fig. 2 ▸) and then back down, but – to avoid any risk of freeing the sample from its pins – we kept a minimal 0.5 N strength corresponding to a 12 µm deflection (number 8). The observed 9 µm difference between deflection numbers 2 and 8 while the strength is identical probably comes from an addition of residual mechanical clearance, backlash and/or layer smoothness under the support pins. We then increased the deflection again, going through the plastic limit. At 31 µm deflection (number 10), a spontaneous plastic stress relaxation occurs, causing the strength to drop from 3.75 to 2.3 N. We continued increasing the deflection until all recorded peaks at any depth clearly displayed a plastic broadening (except at neutral fibre of course). Overall, the deflection range was 0–42 µm, corresponding to a 0–6.2 N strength range, and both are graphically represented in Fig. 2 ▸. Deflections 1 to 9 represent the elastic domain, deflection 10 shows the plastic relaxation, and deflections 11 to 15 are in the plastic domain.

## 4. Data analysis

**Figure 3:**
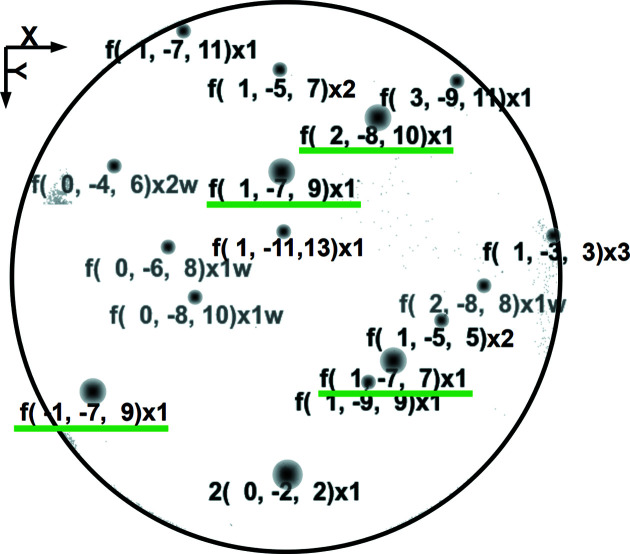
Micro-Laue image showing the position of the 16 main diffraction peaks intercepted by the CCD and their corresponding Miller indices (*h*, *k*, *l*), the four selected peaks being underlined in green. Some supplementary information to Miller indices is also given: before is noted if either the fundamental (‘f’) or only the second harmonic (‘2’) is the lowest available energy peak; after is noted the number *N* of superposed harmonics (‘×*N*’) given the maximum available energy; and eventually a final ‘w’ if the fundamental of this peak is a weak-intensity peak (in light grey).

For this experiment, in order to study intercepted micro-Laue diffraction peaks in fine detail, we have used the same methodology as previously described by Biquard *et al.* (2021 ▸). To summarize, first, the CCD camera is placed sufficiently far away from the sample (∼290 mm) to induce a peak intensity distribution FWHM larger than 3 pixels. Thus, any broadening of diffraction peaks may be precisely recorded. Second, recorded CCD images undergo an overall background removal, making use of a 2D generalized iterative Brückner algorithm. Third, only strong peaks that are not the superposition of different harmonics are considered, and we choose to limit ourselves to the four most intense ones: 177 at 14.2 keV, 1 79 at 16.7 keV, 17 9 at 14.9 keV and 2 8 10 at 16.5 keV, as illustrated in Fig. 3 ▸. The characteristics of these four peaks are averaged together hereafter, and since their energies are close, the corresponding probed volumes are similar. Fourth, we measure precisely the selected peak positions using a purpose-written fitting program that is adapted to the asymmetrical nature of the collected diffraction peaks.

In this experiment, our interest lies in the plastic stress relaxation and, once plastification has begun, the ‘plastification easiness’ (how easy it is to pursue plastification). In a classical HRXRD laboratory experiment, plastification and its associated dislocations induce a 1D peak broadening associated with an intensity drop (Ayers, 1994 ▸). Usually, the FWHM is used as the peak size, just as in Paul Scherrer’s formula (Scherrer, 1918 ▸). But when peaks are asymmetrical, the integral breadth (abbreviated IB hereafter), which is the ratio between the peak area and its maximum, is better suited (von Laue, 1936 ▸; Jones & Bragg, 1938 ▸), taking advantage of a peak area that is independent of the crystallite size. In our case, we deal with asymmetrical 2D peaks: the straightforward idea would be to use for the peak size the IB of the most intense profile in either *X* or *Y*. However, this is a crude simplification, especially as plastification usually leads to inhomogeneous peak broadening along specific directions like easy slip and glide planes (Hull & Bacon, 2011 ▸). As peak broadening may occur in several directions not known beforehand, which may even change under stress, FWHM and IB measurements cannot be fully relevant. To be more consistent, we propose to measure the peak size by generalizing the idea behind the IB. The new 2D IB is defined as the ratio between the peak integral and its maximum (which must always be determined, whether it is for FWHM or IB): the 2D IB is rotationally invariant. And since we are using four peaks at different Bragg angles, dislocations always stay visible, whatever their Burgers vector, since the invisibility criterion (Hirsch *et al.*, 1965 ▸) cannot be satisfied simultaneously for our four peaks. Overall, the 2D IB is perfectly suited to quantify peak size and broadening.

To determine the 2D IB, the most intense pixel is taken as the reference pixel of each peak, and the peak integral is approximated by the sum of the intensities of all pixels situated inside a large 20 pixel radius from the reference pixel, thus accommodating any peak position variation or broadening. Then, a residual local background is calculated as the average intensity of pixels situated in the 25–30 pixel radius range from the reference one, thus compensating for any background left over by the overall background removal. Finally, the peak maximum is precisely determined using a local 2D Gaussian fit that is limited – since peaks are asymmetrical – to the top 5 × 5 pixel zone centred around the reference pixel.

## 5. Results and discussion

### 5.1. 2D IB evolution in the elastic domain

**Figure 4:**
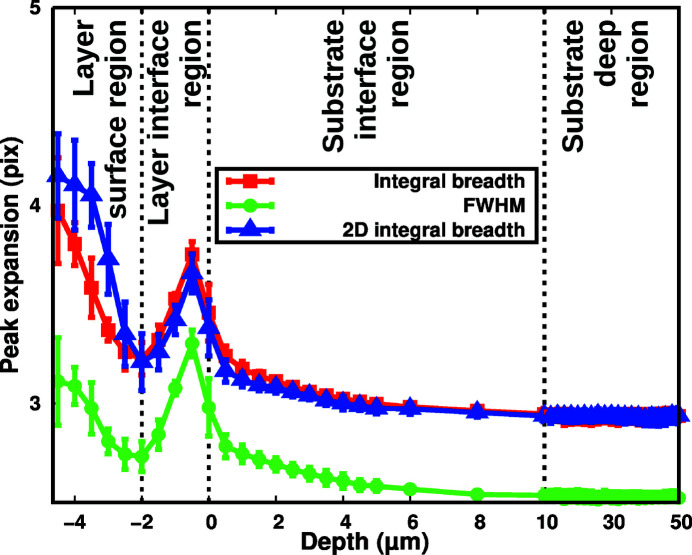
Comparison between the average 2D IB of our four selected diffraction peaks (top blue curve with triangles) and their FWHM (green curve with discs) or IB (red curve with squares) on the elastic domain as a function of depth, whose range was split at depth = 10 µm into two continuous parts of different scale for clarity. The dashed vertical lines at −2, 0 and +10 µm show the limits between the four different regions that may be distinguished inside the sample.

In the elastic domain, we observe that the FWHM, IB and 2D IB show no significant dependency on the deflection value (see the supporting information, Section 7.3), so all peak characteristics were averaged. But FWHM and IB are 1D notions, whereas the 2D IB is a 2D notion. To achieve a meaningful comparison, the FWHM and IB were extended to 2D by using a fixed reference CCD zone for each selected peak. Reference zones were defined so that the reference pixel always stays inside, whatever the deflection or depth, and does not go to the extremities to avoid edge effects. Since peaks are much broader along the growth direction *X* than *Y*, it was chosen as the main direction for FWHM and IB. Finally, zones of 5 pixel height (×30 pixel width) were defined and FWHM and IB were calculated using an area-weighted average of the five slices along *X*. Fig. 4 ▸ compares the evolution of FWHM, IB and 2D IB as a function of depth.

Over the whole substrate range, the ratio between FWHM and IB is found to be remarkably constant at 0.86 ± 0.01, a value that is satisfyingly close to the 0.83 value expected for spherical crystallites after instrumental broadening deconvolution (Langford & Wilson, 1978 ▸). Over an even larger zone (depth ≥ −2 µm), FWHM, IB and 2D IB display the same behaviour, which clearly indicates that the 2D IB is a valid way to measure the peak size. Since the IB is inversely proportional to the volume average of the thickness of the crystallite measured along the normal of the reflecting plane, so is the 2D IB (Stokes & Wilson, 1942 ▸, 1944 ▸).

As shown in Fig. 4 ▸, four different regions may be distinguished as a function of depth. When the X-ray beam probes sufficiently deep inside the substrate – over 10 µm in our case, defining the substrate deep region – we observe that the FWHM, IB and 2D IB stay constant and are minimum (<0.2% variation; a multiplicative factor was applied to the 2D IB in order to match IB). This situation corresponds to a relaxed monocrystalline substrate since the peak shapes are as thin as possible and constant with depth, the final 2D IB corresponding to the Darwin width (Darwin, 1914*a* ▸,*b* ▸) convoluted by all experimental enlargements (CCD ≃ 1.2 pixel point spread function, angular convergence of the X-ray beam at the focal point *etc*.). In the substrate deep region, the X-ray beam probes an undisturbed monocrystalline substrate, showing no influence from the not perfectly matched layer.

As the X-ray beam probes closer to the interface, the 2D IB increases in both layer and substrate, which clearly constitutes the most remarkable feature here. In the 10 µm on the substrate side (defining the substrate interface region), the 2D IB increases linearly by 8 ± 0.15% with decreasing depth. And in a mirror symmetry, it increases by 24.5 ± 0.7% in the 2 µm on the layer side (defining the layer interface region) with increasing depth. This may be interpreted as a micro-strain peak enlargement coming from a strain gradient created by the not perfectly matched layer. Since the strain gradient extension is only 2 µm on the layer side compared with 10 µm on the substrate side, this probably explains the more than three times greater slope. The presence of a strain gradient inside the 2.0 µm-thick layer interface region and in the 10 µm-thick substrate interface region is quite surprising since there is no measurable strain gradient in the tensile case (Biquard *et al.*, 2021 ▸). In the compressive case, a specific mechanism makes the in-plane lattice parameter *a* ^∥^ in the substrate interface region increase when getting closer to the interface in order to accommodate the larger relaxed layer lattice parameter. Since the difference between the *a* ^∥^ of the relaxed layer and that of the substrate is thus lowered compared with the usual not-accommodating-substrate case, this effect may be energetically favoured.

Mismatch is classically measured using HRXRD with an 8 keV X-ray beam incident on the layer side of the sample and only the top few micrometres of the substrate are probed. Mismatch is deduced from the angular difference between layer and substrate symmetrical reflections, that is, from the difference in lattice parameters *a* ^⊥^ measured perpendicularly to the interface, making the assumption that the substrate lattice parameters stay constant with depth. Here, the *a* ^⊥^ of both the substrate and the layer will be smaller in the interface regions than in the usual not-accommodating case. To first order, the two effects will compensate each other, while to second order the difference in Poisson ratio must be taken into account. Consequently, to first order, the difference between the *a* ^⊥^ of the layer and substrate does not depend on whether the substrate accommodates or not and the HRXRD measured mismatch may be considered as correct. Of course, it would be worth assessing the influence of accommodation on the measured mismatch and checking for the absence of a systematic error that could affect a quite large number of measurements, like those given by Ballet *et al.* (2013 ▸). However, this would require a description of the biaxial strain field ε^⊥^ as a function of depth in some detail and then the use of an isotropic elastic modelling (Kisielowski *et al.*, 1996 ▸) to deduce the evolution of *a* ^⊥^, which clearly goes beyond the scope of this article.

Fig. 4 ▸ was obtained by averaging all elastic measurements for which the interface position is determined with a 250 nm (half-depth step) precision. Thus, the effective beam size is the intrinsic 700 nm beam size increased by 250 nm, adding to ∼1.0 µm, that is two depth steps. The minimum 2D IB value observed at depth −2 µm is therefore overestimated, and to estimate it properly we extrapolate using values of the layer interface region, except its extremities, to find 8.6 ± 0.5%. Similarly, in the substrate interface region, we extrapolate the substrate 2D IB at the interface to 8.3 ± 0.1%. Since the two values are almost identical, the critical thickness for the HgCdTe layer with +0.02% compressive mismatch appears to be 2.0 ± 0.25 µm. This seems a reasonable value since the measured critical thickness for the tensile −0.02% mismatch is 2.5 ± 0.3 µm (Biquard *et al.*, 2021 ▸). The critical thickness simply corresponds to the depth value of the minimum of the 2D IB, thus providing an elegant way to measure this fundamental value for any kind of epitaxial layer and even without the need for any flexion machine.

Finally, we define a fourth region named the layer surface region (depth ≤ −2 µm) where the 2D IB significantly increases by 40% when getting closer to the surface. In this region, the 2D IB increases much more than the FWHM or IB. Indeed, the 2D peak shape shows that a supplementary broadening along *Y* exists (see the supporting information, Section 7.4) whose intensity changes with depth, thus making the peak main broadening direction rotate. Such modification of the broadening direction is also present when microfabrication steps like etching, passivation and annealing are used to make operating devices (Tuaz *et al.*, 2017 ▸) (refer to Figs. 3 ▸ and 4 ▸). Overall, the peak broadening direction changes with depth: both FWHM and IB measurements underestimate it but not the 2D IB, since it is by nature independent of the broadening direction. Being beyond the critical thickness, the 2D IB increase shows the presence of misfit dislocations (Yoshikawa, 1988 ▸; Matthews & Blakeslee, 1974 ▸), with an increasing density towards the surface, thus generating this +40% increase.

### 5.2. 2D IB evolution in the plastic domain

Although the 2D IB does not change with deflection in the elastic domain, starting with deflection 10, a spontaneous plastic relaxation occurs and a large peak broadening is observed (the layer surface region is excluded here since it has relaxed even before flexion). At first, it only occurs in the interface layer region (deflection 10), but with subsequent deflection increases, it spreads down into the sample: in half of the substrate interface region with deflection 11 and finally up to 50 µm deep into the substrate with deflection 12. This evolution simply relates to the decrease of the local flexural stress with depth, so we study the 2D IB increase relative to its average elastic value (called the relative broadening) as a function of depth. But depth is not the correct varying parameter, since it is the local flexural stress that triggers plastification.

As the flexion machine applies a uniaxial flexural tensile strain along the *aa* axis of the sample, this will lead to an increase of the peak *Y* position whatever the depth or stress, from which we were able to determine the stress cross-section profile with depth (see the supporting information, Section 7.2; Tuaz, 2017 ▸). In the whole of the substrate, the stress values are found to be linear with depth within  ±1.1%, while in the layer, they are found to be lower than linearly expected (up to −7%) in the interface region and higher (up to 10%) in the surface region. The local flexural stress does not strictly follow a linear variation with depth, but since these variations are small and the uncertainties large, we may consider that the local flexural stress varies linearly with depth.

**Figure 5:**
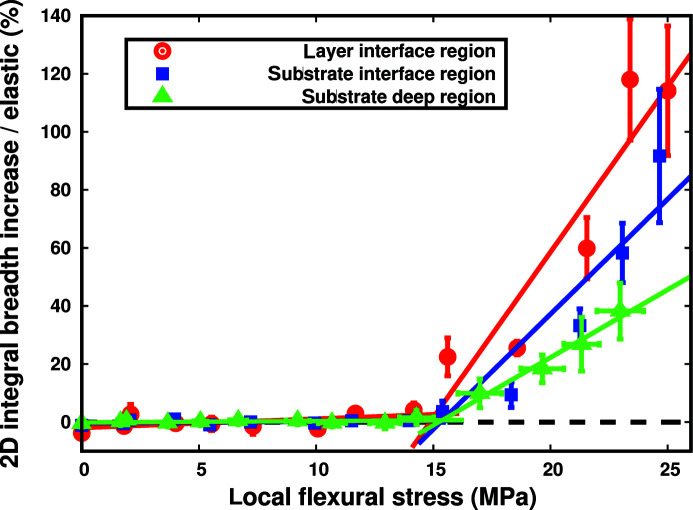
2D IB increase relative to the elastic domain average (see Fig. 4 ▸) for the layer interface region (red curve with round points), the substrate interface region (blue curve with square points) and the substrate deep region (green curve with triangle points) as a function of the local flexural stress. Fits were conducted using all depth values of each region, but for clarity we have only shown here their average value with their standard deviation, while abscissa error bars in fact represent the abscissa range for the substrate deep region. Curves were fitted using a linear fit for both the elastic and the plastic part, and complete results are given in Table 1 ▸.

Finally, we will consider the relative broadening as a function of the local flexural stress for each region separately, as shown in Fig. 5 ▸.

Two linear parts are visible: the first corresponds to the elastic part with an almost constant relative broadening, while the second corresponds to the plastic part with a clear relative broadening increase. Both parts were linearly and independently fitted, and their crossing defines the flexural plastic onset with a 1 MPa precision as shown in Table 1 ▸. With a total variation of the relative broadening in the elastic domain (elastic variability) of less than 5%, averaging data in the elastic domain for Fig. 4 ▸ was legitimate. In the plastic part, the higher the slope, the higher the increase in the 2D IB with stress: this defines a new plastification easiness notion which enables the quantitative evaluation of plastification.

It is important to state that the plastic part would not be linear if we had represented either FWHM or IB as a function of stress because of the rotation of the peak broadening direction (see the supporting information, Section 7.8), thus preventing any precise plastic onset determination and plastification easiness quantification. In contrast, averaging the 2D IB over the critical layer and the substrate regions, we then determine precisely the layer and substrate plastic onsets as well as their plastification easinesses. This novel method is quite general and may be applied to a whole class of epitaxial heterostructures, those for which the critical thickness is simply larger than the micro-Laue beam size (currently 250 nm), an easily validated criterion for quasi-lattice-matched heterostructures.

The most striking feature of Fig. 5 ▸ is that the flexural plastic onset of the three separately considered regions is found to be equal. As our substrate possesses around 4% Zn content, its elastic limit and therefore its plastic onset is expected to be four times that of the layer (Guergouri *et al.*, 1988 ▸). In our case, the substrate plastic onset was significantly decreased to the same value as the layer one. This shows that the dislocations first created inside the layer interface region have threaded through the interface inside the substrate, overcoming the Zn pinning effect (Yoshikawa, 1988 ▸) and thus inducing a plastic transition inside the substrate way below its elastic limit.

We found, for the layer, a flexural tensile elastic limit of 15.1 ± 0.7 MPa, but, from a general point of view, this value is not identical to the tensile elastic limit of the layer. Indeed, the local stress results from the addition of the machine-induced flexural tensile stress along **aa**, and the in-plane compressive mismatch stress that exists independently of flexion. Therefore, our value is an overestimation of the actual tensile elastic limit. Despite this effect, our value is reasonably close to the 12 ± 1 MPa tensile elastic limit determined by a sophisticated in-plane stress experiment using the metric tensor formalism to extract zero-stress lattice parameters (Ballet *et al.*, 2013 ▸). Here, we have only used the 2D IBs, which are an easy way to measure intrinsic peak characteristics to obtain a reasonable value for the elastic limit. A 7 ± 1 MPa in-plane compressive mismatch stress for a +0.02% mismatch was predicted by Ballet *et al.* (2013 ▸), but it was implicitly assumed that the substrate did not accommodate. Here, the substrate accommodation lowers the difference in *a* ^∥^ between the relaxed layer and the substrate compared with the usual not-accommodating-substrate case and therefore the in-plane compressive mismatch stress of the layer is lowered. Our measurement indicates that – keeping 12 MPa as the actual tensile elastic limit – the layer and substrate have roughly equally shared the in-plane mismatch stress, a scenario that is quite plausible.

Although they have the same plastic onset, layer interface and substrate deep regions may be easily differentiated, looking at their plastification easinesses. Indeed, the plastification easiness is 2.4 times higher for the layer than for the substrate, and this is quite a logical finding since the layer is far less rigid than the substrate (Guergouri *et al.*, 1988 ▸). Concerning the substrate interface region – situated between layer interface and deep substrate regions – it may seem at first logical for the plastification easiness to be close to their average. But in this region, we are probing CdZnTe substrate material so that we could expect the same plastification easiness as in the deep substrate region. Indeed, limiting flexural local stress below 22 MPa, we measure a 5.1 ± 0.4% MPa^−1^ plastification easiness, a value close to the substrate deep region’s value. But above this limit around 25 MPa, the plastification easiness increases greatly until it matches that of the layer. Therefore, the substrate interface region presents a transition from a substrate- to a layer-like behaviour in the 22–25 MPa interval. Since dislocations are coming from the layer side, this limits the validity of our assumption to consider the substrate interface region as a whole. This points to the need for a dedicated study where linear fits on the relative broadening are conducted on an individual depth basis with two main improvements: much smaller 50 nm depth steps to follow the beam size shrinking to the current limit of 250 nm as well as more numerous deflection measurements with typically 0.5 µm increments.

### 5.3. Some considerations on 2D IB

One of the strengths of the 2D IB notion is that it completely removes the need to extract any peak profile and that the radii used to define it may be freely chosen as long as they are large enough to accommodate the full peak spreading, smearing, streaking or splitting at the highest studied stress (in our case, 20 pixel radius for integral evaluation and 25–30 pixel radius range for local background). It is therefore quite universal and may be applied to the vast number of *in situ* micro-Laue tensile, compressive, bending or plastic studies (Bhowmik *et al.*, 2016 ▸, 2018 ▸, 2020 ▸; Abboud *et al.*, 2014 ▸; AlHassan *et al.*, 2021 ▸; Kirchlechner, Imrich *et al.*, 2012 ▸).

Still, some limitations exist. The increase in radii will induce a decrease in the measurement precision and the integral evaluation discs cannot overlap. To avoid this intrinsic limitation – in the case of notable streaking in peaks (Jun *et al.*, 2022 ▸) for example – instead of a disc, it may be worth choosing a rectangle orientated along the average streak direction, provided the width is sufficient to accommodate peak rotation with stress. Also, integral discs are logically centred on the position of the maximum, but this is not a requirement at all. Indeed, when peaks are so smeared out that they do not really possess a centre and are more blob like, the position of the maximum may bounce around with stress (Kirchlechner, Grosinge *et al.*, 2012 ▸) and it may be best to use fixed-position integration discs. Another limitation arises from the need to determine the maximum intensity of the peak. This is especially difficult when peaks split into several components that drift apart with stress (Schneider *et al.*, 2012 ▸). To stay coherent in such a case, the peak maximum should be defined as the sum of the local maximum of each component since, before splitting, components were superimposed.

To enable comparison between different materials (CdZnTe substrate and HgCdTe layer here), the plastification easiness is deduced from the slope of 2D IB increase relative to the average elastic value. Therefore, the elastic 2D IB has to be measured and this may appear difficult for layers that display an even lower elastic limit than our 10–20 MPa. In any case, it is always possible to simply use the 2D IB value measured without any additional stress, that is with the sample free-standing. This measurement may also provide the sample critical thickness as the minimum position of the 2D IB.

An (approximate) elastic limit is deduced from the intercept of the linear fit of the 2D IB increase relative to the elastic value. To achieve this, we only need to record data on a limited range of stress values in the plastic domain, not on the fully available range. Therefore, even in the case of peaks with large spreading, smearing, streaking or splitting, we may keep reasonable radii and therefore avoid all limitations.

## 6. Conclusion

The new 2D IB notion was successfully used to characterize a quasi-lattice-matched HgCdTe/CdZnTe heterostructure as a function of the local flexural stress induced by a flexion machine. Cross-section profiles were recorded using micro-Laue diffraction, which showed that plastification induces a large peak broadening whose main direction changes with both depth and stress. Usual FWHM or IB values are therefore no longer relevant and only the rotationally invariant 2D IB – defined as the ratio between the peak integral and its maximum – correctly measures plastification-induced peak broadening.

Cross sections showed that the sample must be divided according to depth into four different regions that behave quite differently. Sufficiently deep inside, the substrate is logically found to be undisturbed by the layer with a constant and minimal 2D IB. A region situated 10 µm beneath the interface displays a 2D IB increase interpreted as an in-plane lattice adjustment to the +0.02% mismatched layer, which is equally shared with the layer. The CdZnTe substrate is found to be deformed in the vicinity of the interface in the case of a compressive mismatch of the HgCdTe layer, not in the tensile case. The 2D IB minimum indicates a 2 µm critical thickness for the HgCdTe layer, and beyond, a large peak broadening occurs as a result of the presence of misfit dislocations. Taking into account only the critical thickness region, we measured a 15.1 ± 0.7 MPa tensile flexural elastic limit for HgCdTe, which is slightly overestimated compared with the elastic limit. Although the substrate elastic limit is expected to be four times higher, the CdZnTe substrate starts plastification at the same 15.1 MPa value, showing that the layer dislocations have threaded through the interface. Once plastification has started, in the critical thickness layer region, the plastification easiness is 2.4 times higher than it is deep inside the substrate, while in the lattice adjustment 10 µm region, it increases from the substrate to the layer with a 22–25 MPa transition interval. Because it displays both a lattice adjustment and a plastification easiness transition, the substrate 10 µm region situated below the interface would be worth a more thorough investigation combining both strain and 2D IB measurements using today’s smaller beam size. This new method using the 2D IB is quite general since it may be applied to the vast class of epitaxial layers for which the critical thickness is simply larger than the micro-Laue beam size (250 nm nowadays), and allows for easy critical thickness measurement as well as precise plastic onset determination and plastification easiness assessment.

## Supplementary Material

Supporting information. DOI: 10.1107/S1600576722008184/ei5083sup1.pdf

## Figures and Tables

**Table 1:** Results of the two-part linear fits shown in Fig. 5 ▸ The flexural plastic onset corresponds to the abscissa of the crossing between the two linear fits, the plastification easiness corresponds to the slope of the plastic fit and the elastic variability is the total variation of the elastic fit from 0 to the flexural plastic onset.

Region	Layer interface	Substrate interface	Substrate deep
Flexural plastic onset (MPa)	15.1 ± 0.7	15.4 ± 1.4	15.4 ± 0.3
Plastification easiness (% MPa^−1^)	11.4 ± 0.9	8.8 ± 0.4	4.7 ± 0.2
Elastic variability (%)	4.6 ± 7.7	1.0 ± 0.4	0.8 ± 0.8
