# Supplementary material for: Using 2D integral breadth to study plastic relaxation in a quasi-lattice-matched HgCdTe/CdZnTe heterostructure
Source: J Appl Crystallogr. 2022 Oct 1;55(Pt 5):1297–304. doi: 10.1107/S1600576722008184 (PMC9533753; doi:10.1107/S1600576722008184)
Supplement: Supplementary file 1 [file j-55-01297-sup1.pdf]

7 Supplementary material

7.1 Study of the test sample

The flexion machine was working in constant deflection mode where the sample (central) deflection is adjusted and kept constant thanks to an optical 20 nm resolution encoder, while strength evolution on the load pin is measured. Fig. 6a shows the results on the test sample, where we have used four successive large steps in the deflection to go from the elastic to the plastic domain of the HgCdTe layer. We logically observe that strength grows with deflection but in a clearly non-proportional way with two important characteristics. The first characteristic relates to the first step that goes from 0 to 36 µm deflection: despite it is 5 times bigger than the three following ones, it induces a 3 times smaller strength increase. This indicates a large 20-25 µm deflection offset in our 'zero' of the deflection. Consequently, for the data sample and prior to any measurement, several back-and-forth cycles at low strengths were conducted to allow for a good positioning of supporting pins on the sample and to accommodate as much as possible for any mechanical clearance or backlash.

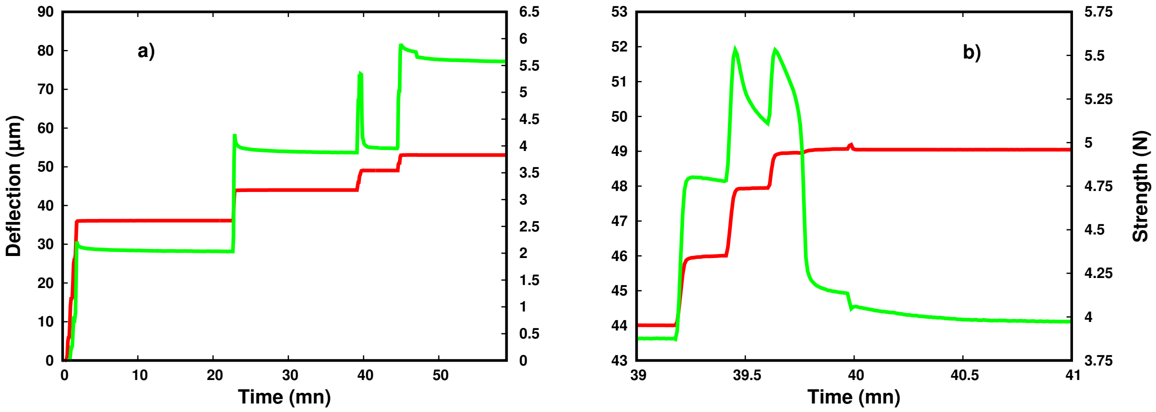

Figure 6: a): Strength evolution (green curve — right axis) with time during the four successive large steps in deflection (red curve — left axis) on the test sample taken at 36, 44, 49 and 53 µm. b): Detailed view of the elastic to plastic transition occurring between a deflection of 44 and 49 µm and the associated large spontaneous strength relaxation.

The second characteristic occurs at the third step going from 44 to 49 µm deflection as shown in details in Fig. 6b. To rise from 44 to 49 µm, we have taken two intermediate values at 46 and 48 µm. Strength does increase normally during the first intermediate value, but already shows some relaxation for the second one. And when finally reaching 49 µm – after an expected increase in strength – a spectacular spontaneous decrease occurs going from 5.5 N down to 4.0 N before stabilizing at 3.9 N! This evidences the plastic relaxation of the HgCdTe layer, since it undergoes a maximal flexural stress and has a 4 times lower elastic limit than the substrate [1]. As we must be sensitive to any spontaneous drop in strength because this will sign the elastic to plastic transition, the flexion machine cannot work in the usual constant strength mode but rather in the constant deflection mode. And once a deflection step has been taken, we will wait between 2 and 3 minutes for the strength to stabilize before recording the cross-section profile.

7.2 Stress cross-section profile with depth

The flexion machine applies an uniaxial flexural tensile strain along the $\vec{aa}$ axis of the sample and consequently, while in the elastic domain, the sample undergoes a compressive strain along the $\vec{bb}$ and $\vec{cc}$ axes, the proportionality ratio being the Poisson ratio by definition. The Poisson ratio along the $\vec{cc}$=[211] being close to 1/3 (0.323, refer to [2] equation 1.19), the main effect in plane (X,Y) will be tensile along axis $\vec{Y} = \vec{aa}\cos(40°) + \vec{bb}\sin(40°)$. The diffraction planes will reduce their angle with the X-ray beam in the (Y,Z) plane, leading to an increase of peak Y position whatever the depth or stress as shown in Fig. 7. The maximum 1.2/100° angle reduction is negligible compared to the total angular deviation 2θ which is in the 90±15° range and therefore the product between the 2D integral breadth and the crystallite size remains constant. Finally, in the elastic domain, only the peak position depends on the stress, not the 2D integral breadth.

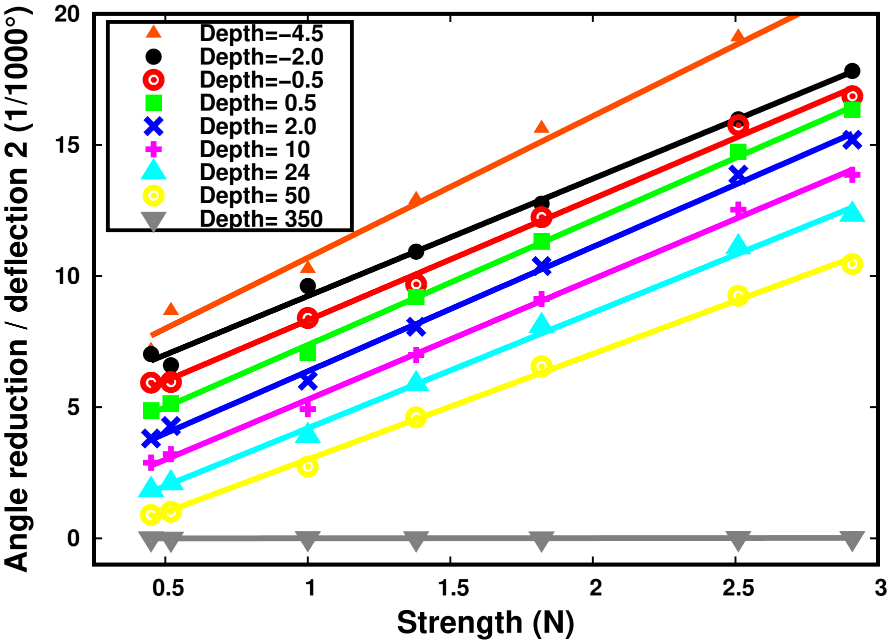

Figure 7: Angle reduction of the diffraction planes with the X-ray beam in the (Y,Z) plane deduced for the Y peak position increase relative to deflection 2 in the elastic domain (deflections 2 to 8), as a function of strength for various depths covering all four sample regions. For each depth, linear regressions are shown, displaying different slopes (a unity offset was used between successive depths for clarity)

It must be emphasized here that failure to compensate the sample movements induced by the mechanical strain inside the flexion machine will dominate the Y position changes and render this effect invisible. Indeed, when deflection changes, mechanical strain inside the flexion machine changes, and this will inevitably induce small spatial translations and rotations of the sample relative to the X-ray beam and CCD. These sample movements must be compensated for and to this end, we use neutral fibre CCD images since they are independent of deflection. Because sample movements are small, we cannot distinguish between sample translation and rotation along X or Y: we will simply maintain constant the average position of our four selected peaks. Between successive deflections, we find small average position changes along X of $\pm 128\,\mu$m (1.6 pixels) corresponding to 2.5/100°

and along Y of $\pm 74\,\mu m$ (0.9 pixel) corresponding to $1.5/100°$. The only large (10 pixels) average position change occurs between the first deflection and the second: at 'zero' strength, the sample is free to move between pins and does so. Once the X and Y movements are compensated, we assess Z movements using individual peak positions in the polar coordinate system. We find a small Z rotation of $\pm 1.3/100°$ and a very small $\pm 12.4\,\mu m$ translation compared to the $\approx$290 mm sample distance to CCD. With all movements compensated, the residual variations of individual diffraction peak positions at the neutral fibre for non-zero strength deflections (2 to 15) are extremely low since they are under $6.3\,\mu m$ (0.08 pixel) corresponding to $\pm 1.2/1000°$. After compensation, the residual sample movements induced by deflection changes are found to be quite small and ten times smaller than the angle reduction of the diffraction planes.

Fig. 8 shows the evolution of the slope of the angle reduction as a function of depth. As slope is proportional to the local flexural stress, Fig. 8 is in fact a stress cross-section profile with depth, a quite valuable information. With a monocrystalline sample, the local flexural stress would decrease linearly with depth from the surface where it is maximal, until the neutral fibre where it is always zero. This is observed in the whole substrate with experimental values within $\pm$1.1% from the linear fit. In the layer, values are lower than linearly expected (up to -7%) in the interface region, while higher (up to 10%) in the surface region. The local flexural stress does not strictly follow a linear variation with depth, but these variations being small and uncertainties large, we will consider that the local flexural stress varies linearly with depth.

7.3 Unchanged peak characteristics in the elastic domain

As said in 5.1, there is no significant dependency with the deflection value of FWHM, IB and 2D integral breadth on the elastic domain. This is illustrated on Fig. 9.

7.4 Peak broadening and rotation increase when closing to the surface in the layer surface region

In the layer surface region (see 5.1) which possesses misfit dislocations even in the absence of any supplementary flexural stress, a clear peak broadening and rotation occurs when closing to the surface, as is illustrated in Fig. 10.

7.5 Peak broadening and rotation increase with deflection in the plastic domain

The rotation of the peak broadening direction as a function of stress is illustrated in Fig. 11. As said in 5.2, if FWHM or IB were represented as a function of stress, the plastic part of Fig.$\tilde{5}$ would not be linear, thus preventing any precise plastic onset determination and plastification easiness quantification. This is illustrated in Fig. 12 where we compare, for the layer critical thickness region, peak size as measured by 2D integral breadth with IB (along X) as a function of stress. We observed that *specifically in our case here*, all four peaks broaden for deflection 11 roughly along X before broadening for deflections 12 to 15 roughly along Y. So, to be complete and thorough, we have also represented the IB taken along the Y direction as well as the sum of IB along X and Y since it should be almost representative of this two successive X and Y broadenings. And indeed, the sum

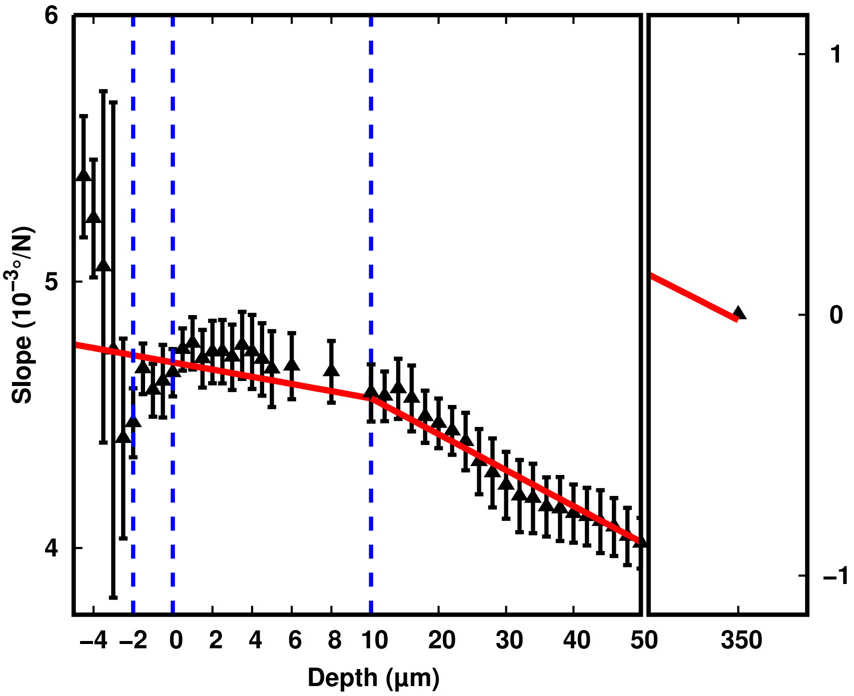

Figure 8: Slope (refer to Fig. 7) of the angle reduction of the diffraction planes with the X-ray beam in the (Y,Z) plane in the elastic domain (deflections 2 to 8) as a function of depth (black triangular points). Abscissa range was split in three parts: the left part corresponds to the layer and the substrate interface regions at a high scale, the middle part represents the substrate deep region until 50 µm at a lower scale and the third shows the neutral fibre. Linear regression of the substrate deep region (depth≥10 µm) is shown in red while blue dashed vertical lines at -2, 0 and +10 µm show the region limits.

looks like the 2D integral breadth evolution but still, using the 2D integral breadth to measure the peak size makes more sense since it is rotationally invariant.

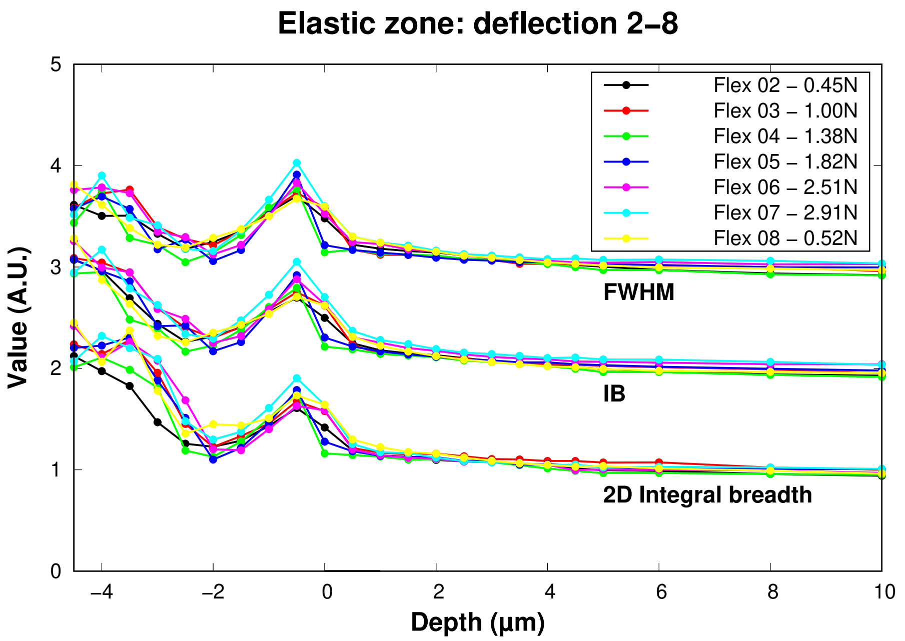

Figure 9: Individual evolution of 2D integral breadth, IB and FWHM as a function of depth for all deflections of the elastic domain. For clarity's sake, all graphs were offsetted from one another using the [5-10] µm average value. This value was fixed at 1 for the 2D integral breadth, 2 for the IB and 3 for the FWHM.

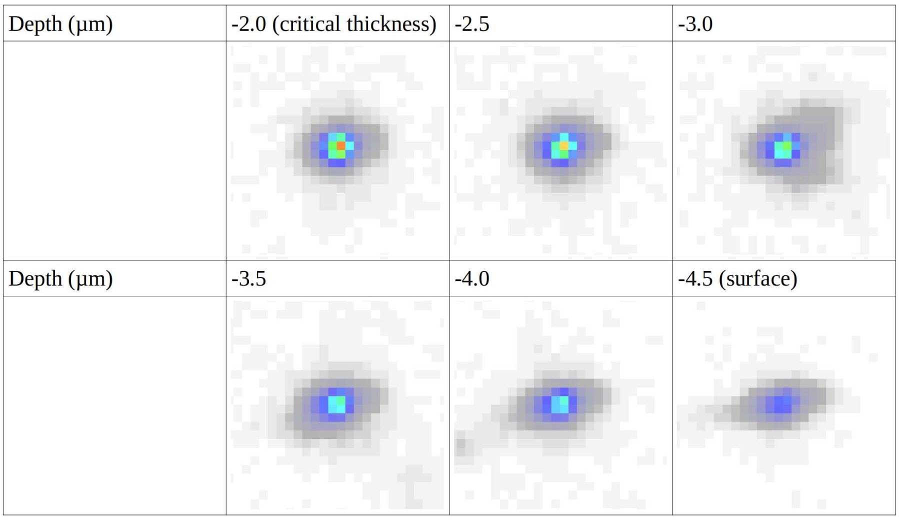

Figure 10: Evolution of the (1,-7,9) diffraction peak collected at deflection number 6 (2.51 N) as a function of depth on the layer surface region. The peak broadening increases clearly when closing to the surface while it also rotates. The same colour scale was used to represent the intensity.

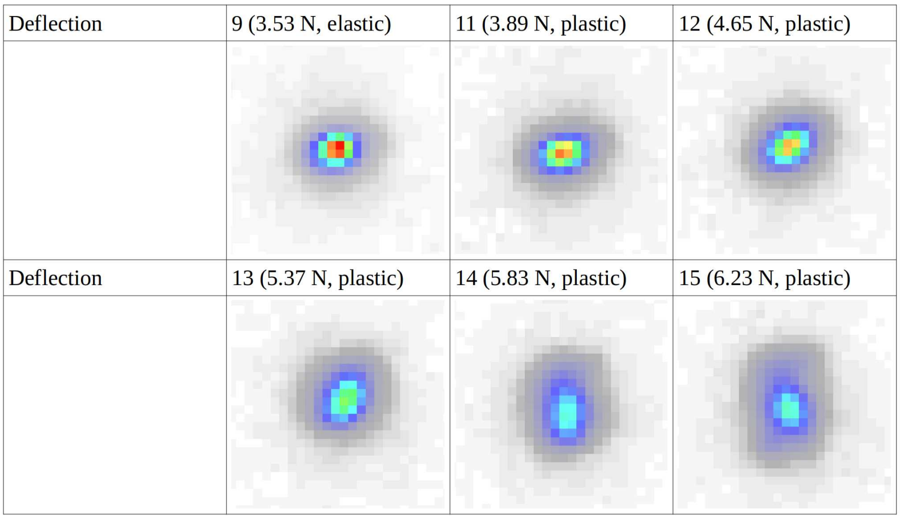

Figure 11: Evolution of the (-1,-7,9) diffraction peak collected at depth -1 µm (centre of the layer interface region) as a function of deflection number 10-15. The peak broadening along Y is clearly visible when entering the plastic domain (deflections 11-15) inducing a clear rotation with deflection. The same color scale was used to represent the intensity.

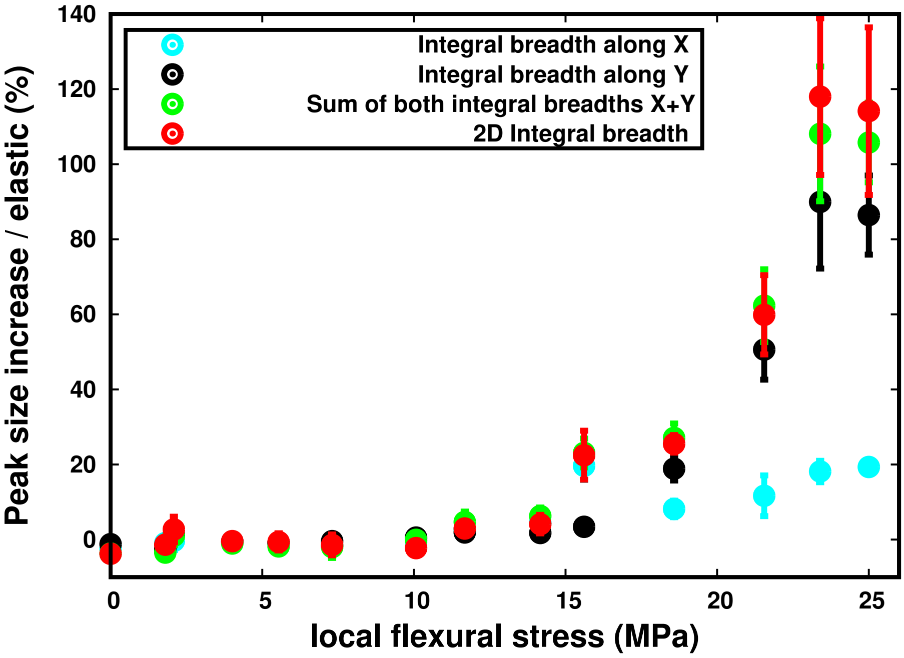

Figure 12: Comparison between integral breadth values taken along X or Y as well as their sum with the 2D integral breadth as a function of flexural stress on the layer interface region

References

[1] K. Guergouri, R. Triboulet, A. TromsonCarli, and Y. Marfaing. Solution hardening and dislocation density reduction in CdTe crystals by Zn addition. *Journal of Crystal Growth*, 86(1):61–65, January 1988.

[2] Aymeric Tuaz. *Investigations Structurales Haute-Résolution de Photodiodes Infrarouges de Nouvelle Génération.* PhD thesis, Université Grenoble Alpes - CEA/Grenoble - http://www.theses.fr/s119377, Grenoble, December 2017.
